# Stunting and severe stunting among children under-5 years in Nigeria: A multilevel analysis

**DOI:** 10.1186/s12887-016-0770-z

**Published:** 2017-01-13

**Authors:** Blessing Jaka Akombi, Kingsley Emwinyore Agho, John Joseph Hall, Dafna Merom, Thomas Astell-Burt, Andre M. N. Renzaho

**Affiliations:** 1 School of Science and Health, Western Sydney University, Locked Bag 1797, Penrith, NSW 2571 Australia; 2 School of Medicine and Public Health, Faculty of Health, University of Newcastle, Callaghan, NSW 2308 Australia; 3 Population Wellbeing and Environment Research Lab (PowerLab) School of Health and Society, Faculty of Social Sciences, University of Wollongong, Wollongong, NSW 2522 Australia; 4 Early Start Research Institute, Faculty of Social Sciences, University of Wollongong, Wollongong, NSW 2522 Australia; 5 Illawarra Health and Medical Research Institute, Wollongong, NSW 2522 Australia; 6 School of Social Sciences and Psychology, Western Sydney University, Locked Bag 1797, Penrith, NSW 2751 Australia

**Keywords:** Stunting, Severe stunting, Nigeria, Public Health, Malnutrition, Multilevel analysis

## Abstract

**Background:**

Stunting has been identified as one of the major proximal risk factors for poor physical and mental development of children under-5 years. Stunting predominantly occurs in the first 1000 days of life (0–23 months) and continues to the age of five. This study examines factors associated with stunting and severe stunting among children under-5 years in Nigeria.

**Methods:**

The sample included 24,529 children aged 0–59 months from the 2013 Nigeria Demographic and Health Survey (NDHS). Height-for-age z-scores (HFAz), generated using the 2006 World Health Organisation (WHO) growth reference, were used to define stunting (HFAz < −2SD) and severe stunting (HFAz < −3SD). Multilevel logistic regression analyses that adjusted for cluster and survey weights were used to determine potential risk factors associated with stunting and severe stunting among children under-5 years in Nigeria.

**Results:**

The prevalence of stunting and severe stunting were 29% [95% Confidence interval (Cl): 27.4, 30.8] and 16.4% [95%Cl: 15.1, 17.8], respectively for children aged 0–23 months, and 36.7% [95%Cl: 35.1, 38.3] and 21% [95%Cl: 19.7, 22.4], respectively for children aged 0–59 months. Multivariate analysis revealed that the most consistent significant risk factors for stunting and severe stunting among children aged 0–23 months and 0–59 months are: sex of child (male), mother’s perceived birth size (small and average), household wealth index (poor and poorest households), duration of breastfeeding (more than 12 months), geopolitical zone (North East, North West, North Central) and children who were reported to having had diarrhoea in the 2 weeks prior to the survey [Adjusted odds ratio (AOR) for stunted children 0–23 months = 1.22 (95%Cl: 0.99, 1.49)];[AOR for stunted children 0–59 months = 1.31 (95%Cl: 1.16, 1.49)], [AOR for severely stunted children 0–23 months = 1.31 (95%Cl: 1.03, 1.67)]; [AOR for severely stunted children 0–59 months = 1.58 (95%Cl: 1.38, 1.82)].

**Conclusions:**

In order to meet the post-2015 sustainable development goals, policy interventions to reduce stunting in Nigeria should focus on poverty alleviation as well as improving women’s nutrition, child feeding practices and household sanitation.

## Background

Stunting is a major health problem in children under-5 years in many low and middle income countries around the world [1]. It is defined as a deficit in height relative to a child’s age [2]. Stunting in children under-5 years could lead to impaired physical development and have a long-term effect on cognitive development, educational performance and economic productivity in adulthood and on maternal reproductive outcomes [3].

There is a global agreement on a critical window—from conception through the first 2 years of life (0–23 months)—within which 70% of stunting occurs [4]. This linear growth deficit continues to deteriorate till the age of five due to sustained exposure to unpleasant environmentally modifiable factors related to feeding, infections and psychosocial care [4]. The continued decline in linear growth observed in the first 5 years of life may cause severe irreversible physical and neurocognitive damage that accompanies stunted growth and pose a major threat to human development.

Importantly, stunting is a major contributor to child morbidity and mortality; thus providing evidence for more effective policies and programs to prevent child undernutrition, and its associated lifelong disabilities is crucial in achieving the global nutrition targets for 2025 which was adopted by the World Health Assembly [5], and has been proposed as a leading indicator for the post-2015 development agenda.

Sub-Saharan Africa and South Asia have reported the highest prevalence of stunted children in the world. In sub-Saharan Africa, 37% of children under-5 years are stunted [1]. A comparison of regional trends in the number of children affected by stunting in sub-Saharan Africa further revealed an increase from 20 million children in 1990 to 28 million children in 2013 [6]. Also, available data from the United Nations Children’s Fund estimates the prevalence of childhood stunting in Nigeria at about 36% in 2013 which indicates that stunting remains a major public health problem in the country just like in many other developing countries [1].

Past studies conducted in Ogun State [7], Osun State [8] and in the middle belt of Nigeria [9] on factors associated with stunting indicated that maternal education, wealth index, duration of breastfeeding and presence of infections were associated with stunting. The major limitations of these studies were that, factors associated with severe stunting (HFAz < −3SD World Health Organisation growth reference) were not examined and the studies were regional. Hence, their findings could not be generalised to the wider Nigerian population. In 2013, a population-based study was conducted using data from the 2008 Demographic and Health Survey (DHS). The study analysed the variations in prevalence of child stunting in various states of Nigeria, but was limited by the fact that the authors failed to adjust potential confounding factors [10].

In order to improve child nutrition in Nigeria, a population-based study with sufficient sample size is needed to provide a comprehensive understanding of the factors associated with stunting and severe stunting. Hence, the main aim of this study is to utilise data from 2013 Nigeria Demographic and Health Survey (NDHS) to determine the factors significantly associated with stunting and severe stunting among children aged 0–59 months after controlling for potential confounding factors. Findings from this study can be generalised to populations with similar characteristics and would be useful to policy makers and public health researchers in formulating effective interventions aimed at reducing the number of stunted and severely stunted children by strategically targeting the most vulnerable subpopulations.

## Method

### Data source

The dataset used in this study was obtained from 2013 NDHS. The survey was implemented by the National Population Commission (NPC) in conjunction with ICF Macro, Calverton, MD, USA [11].

A representative sample of 40,680 households was selected for the survey, with a minimum target of 943 completed interviews per state. A stratified three-stage cluster design consisting of 904 clusters, 532 in rural areas and 372 in urban areas was used in the selection of samples for the survey. For each cluster, a fixed sample of 45 households was selected. All women aged 15–49 who were either permanent residents of the households in the 2013 NDHS sample or visitors present in the households on the night before the survey were eligible to be interviewed.

In the interviewed households, 39,902 women aged 15–49 were identified as eligible for individual interviews and 98% of them were successfully interviewed. A total of 38,948 women were interviewed of which 15,545 resided in urban areas and 23,403 in rural areas. Structured survey questionnaires were used in the interviews to collect relevant information on the respondent’s demographics, socio-economic status, anthropometry as well as maternal and childcare practices.

A total of 30,050 children under the age of five were eligible for anthropometric measurements. There was an overall 96% response rate for children with respect to height measurements. 88% of the measurements carried out for children were valid. Hence, the following analysis focuses on the 24,529 children with valid and complete information on date of birth and height (in centimetres) [11].

### Dependent Variables: Stunting and Severe Stunting (Height-for-age)

Measurements of height were obtained for children under the age of five preceding the survey in all of the selected households. Each team of interviewers carried a weighing scale and measuring board. Measurements were made using lightweight SECA scales (with digital screens) designed and manufactured under the authority of the United Nations Children’s Fund (UNICEF). The measuring boards employed were specially made by Shorr Productions for use in survey settings. Children under the age of 2 were measured lying down on the board (recumbent length), and standing height was measured for older children.

The height-for-age index of children was calculated using growth standards published by the World Health Organization (WHO) in 2006. These growth standards were generated through data collected in the WHO Multicentre Growth Reference Study [2] and expressed in standard deviation units from the Multicentre Growth Reference Study median. The height-for-age index is an indicator of linear growth retardation and cumulative growth deficits in children. Children with height-for-age Z-score below minus two standard deviations (−2 SD) from the median of the WHO reference population are considered to be stunted or chronically malnourished while children who are below minus three standard deviations (−3 SD) from the reference median are considered severely stunted.

### Descriptive Study Variables

**Fig. 1:**
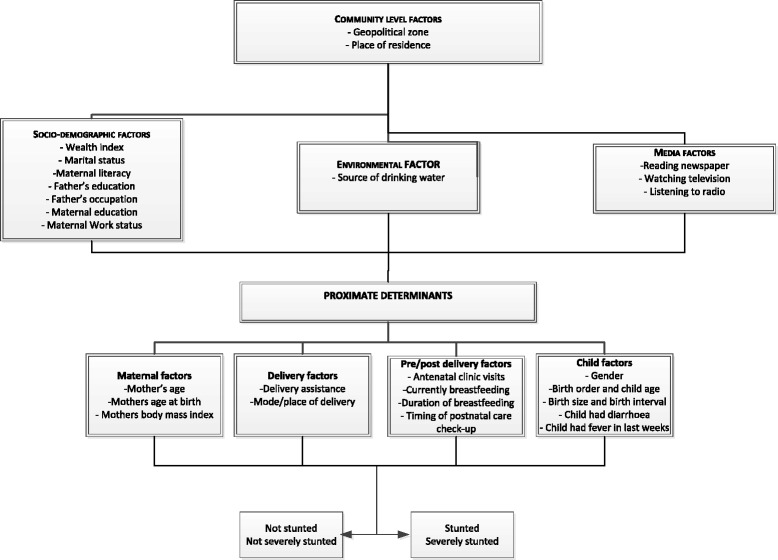
Conceptual framework for analysing factors associated with stunting and severe stunting in children under-5 years in Nigeria. Adapted from UNICEF Conceptual Framework (2013)

The potential risk factors were classified into five main categories which include: Community level factors, socio-demographic factors, environmental factors, media factors and proximate determinants as shown in Fig. 1.

Community level factors included geopolitical zone and type of residence. Geopolitical zones were defined based on ethnic homogeneity of near-perfect political, administrative and commercial city in Nigeria. The socio-demographic factors included wealth index, marital status, maternal literacy, paternal education, paternal occupation, maternal education and maternal work status. Household wealth index serves as an indicator of wealth that is consistent with expenditure and income measures. It was represented as a score of household assets via the principle components analysis method (PCA) [12]. Once this index was computed, scores were assigned to each de jure household member, ranking each person in the population by his or her score. The index was categorized into five national-level wealth quintiles: poorest, poor, middle, rich and richest. The bottom 40% of the households was referred to as the poorest and poor households, the next 20% as the middle-class households, and the top 40% as rich and richest households.

The environmental factor was source of drinking water which was categorized into improved and unimproved according to WHO/UNICEF guidelines [13]. Media factors were reading of newspaper, watching television and listening to radio. The proximate determinants included maternal factors, delivery factors, pre/post-delivery factors and child factors. Maternal factors were mother’s age, mother’s age at birth and mother’s body mass index. Delivery factors included place of delivery, mode of delivery and type of delivery assistance. A combination of place of delivery and mode of delivery was subdivided into three categories: home delivery, delivery at health facility with non-caesarean section and delivery at health facility with caesarean section. Pre/post-delivery factors included antenatal clinic visits, timing of postnatal care check-up, currently breastfeeding, and duration of breastfeeding. Child factors included sex, birth order, child’s age in months, perceived birth size, preceding birth interval, child had diarrhoea and had fever 2 weeks preceding the survey.

### Statistical Analysis

To determine factors associated with stunting and severe stunting in children aged 0–23 months and children 0–59 months, the dependent variables were expressed as a dichotomous variable, i.e. category 1 [stunted (> − 2SD) or severely stunted (> − 3SD)] and category 0 [not stunted (> − 2SD) or not severely stunted (> − 3SD)].

Analyses were performed using Stata version 14.0 (StataCorp, College Station, TX, USA). The Taylor series linearization method was used in the surveys to estimate the confidence intervals (Cls) around prevalence estimates of stunting and severe stunting amongst children aged 0–23 months and 0–59 months. Generalized linear latent and mixed models (gllamm) with the logit link and binomial family [14] were used to determine the factors significantly associated with stunting and severe stunting.

The multivariable analysis conducted used a five-staged conceptual modelling technique (see Fig. 1). In the first stage, community level factors were entered into the baseline model to assess their association with the study outcomes. A manual stepwise backward elimination method was conducted and factors significantly associated with the study outcomes were retained. In the second modelling stage, socio-demographic factors were added to the significant factors from the first model and a backward elimination procedure was performed. This approach was repeatedly used for the inclusion of environmental factors, media factors and proximate determinants in the third, fourth, and fifth stages respectively. In each stage, the factors with *p*-values <0.05 were retained. To avoid any statistical bias, we double checked our results by: (1) entering only potential risk factors with a *p*-value < 0.20 obtained in the univariable analysis for backward elimination process, (2) testing the backward elimination method by including all of the variables (all potential risk factors), and (3) Collinearity was tested and reported in the final model. The adjusted risk of independent variables was assessed by calculating the odds ratios with 95%Cls and those with *p* < 0.05 were retained in the final model.

## Results

### Characteristics of the sample

A total sample of 24,529 children aged 0–59 months was included in the study. Of these, 37% lived in urban areas while 63% lived in rural areas. Male (49.7%) and female (50.3%) children were almost equally represented. About 12.4% of mothers had visited the antenatal clinic at least once during pregnancy and 42.8% had delivery assistance from a health professional. Approximately 22% was in the poorest and 18% in the richest wealth index quintile. See Table 1 below.

**Table 1:** Characteristics of risk factors associated with stunted and severely stunted children aged 0–59 months in Nigeria 2013

Characteristics	Number	Percent
*Community level factors*		
Type of residence		
Urban	9067	37.0
Rural	15465	63.0
Geopolitical Zones		
North Central	3562	14.5
North East	4086	16.7
North West	8506	34.7
South East	2284	9.3
South West	2372	9.7
South South	3722	15.2
*Socio*-*demographic factors*		
Maternal working status		
Non-working	16151	97.1
Working (past 12 months)	485	2.9
Maternal education		
No education	11378	46.4
Primary	4933	20.1
Secondary and above	8221	33.5
Father’s occupation		
Non agriculture	20237	82.5
Agriculture	1024	4.2
Not working	3271	13.3
Father’s education		
No education	8870	37.0
Primary	4640	19.4
Secondary and above	10447	43.6
Marital status		
Currently married	23592	97.6
Formerly married (div/sep/widow)	579	2.4
Mother’s literacy		
Can’t read at all	14029	57.5
Can read	10386	42.5
Wealth Index		
Poorest	5378	21.9
Poor	5383	21.9
Middle	4711	19.2
Rich	4598	18.7
Richest	4462	18.2
*Environmental factor*		
Source of drinking water		
Improved	13878	56.6
Unimproved	10653	43.4
*Media factors*		
Reading newspaper		
Yes	3589	14.7
No	20793	85.3
Listening to radio		
Yes	15135	61.9
No	9314	38.1
Watching television		
Yes	11690	47.9
No	12732	52.1
*Proximate determinants*		
*Maternal factors*		
Mother’s age		
15–24 years	5780	23.6
25–34 years	12424	50.6
35–49 years	6328	25.8
Mother’s age at birth		
< 20 years	3325	13.6
20–29 years	12878	52.5
30–39 years	7161	29.2
40 and above	1168	4.8
*Delivery factors*		
Type of delivery assistance		
Health professional	10399	42.8
Traditional birth attendant	4938	20.3
Relatives and other untrained personnel	5856	24.1
No one	3113	12.8
Place of delivery		
Home	15065	61.4
Health facility	9467	38.6
Mode of delivery		
Non-caesarean	23734	97.8
Caesarean	523	2.2
Combined Place and mode of delivery		
Non-caesarean and Home delivery	15065	62.1
Non-caesarean & Health facility	8669	35.7
Caesarean & Health facility	523	2.2
*Pre*/*Post*-*delivery factors*		
Antenatal clinic visits		
None	5177	32.8
1–3.	1954	12.4
4 and above	8674	54.9
Timing of postnatal check-up		
No postnatal check-up	19243	78.4
0–2 days	3748	15.3
Delayed	1541	6.3
Currently breastfeeding		
Yes	13950	56.9
No	10582	43.1
Duration of breastfeeding		
Upto 12 months	5376	22.3
> 12 months	18792	77.8
*Child factors*		
Birth order		
First-born	4641	19.0
2nd-4th	11327	46.2
5 or more	8564	34.9
Preceding birth interval		
No previous birth	4641	19.0
< 24 months	4326	17.7
> 24 months	15520	63.4
Gender		
Male	12193	49.7
Female	12339	50.3
Perceived size of baby		
Small	3385	14.0
Average	10052	41.5
Large	10759	44.5
Child’s age in months		
0–5	2238	9.3
18–23	2130	8.9
24–29	2496	10.4
30–35	2105	8.8
36–41	2763	11.5
42–47	2124	8.8
48–53	2488	10.4
54–59	1939	8.1
Child had diarrhoea recently		
No	21885	89.3
Yes	2556	10.7
Child had fever in last 2 weeks		
No	21251	86.6
Yes	3153	13.4

**Fig. 2:**
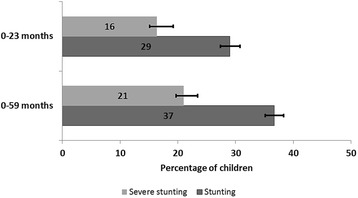
Prevalence of stunting and severe stunting in children under-5 years in Nigeria

Figure 2 shows the prevalence of stunting and severe stunting. The figure reveals a statistical significant difference between stunting for children aged 0–23 and 0–59 months whereas, severe stunting for children aged 0–23 and 0–59 months did not differ statistically.

### Factors associated with stunting

#### Children aged 0–23 months

In Table 2, children born to uneducated fathers were more likely to be stunted when compared with children born to educated fathers. Male children were more likely to be stunted when compared with their female counterpart. Babies perceived by their mothers to have been small at birth were more likely to be stunted than children perceived to have been large. Children who were breastfed for more than 12 months were more likely to be stunted when compared with children breastfed for less than 12 months. Children from the poorest households were more likely to be stunted than children from the richest households. Children who reside in the North West geopolitical zone were more susceptible to stunting than children who reside in other geopolitical zones. Also, children who had a recent episode of diarrhoea were more likely to have stunted growth when compared with children who had not. Increasing age of the child was significantly associated with stunted growth.

**Table 2:** Unadjusted and adjusted odds ratios (OR) (95% CI) for stunted children aged 0–23 and 0–59 months

Characteristic	Stunted children 0–23 Months				Stunted children 0–59 Months			
	Unadjusted Odd Ratio (OR) [95%CI]	*P*	Adjusted Odd Ratio (AOR) [95%CI]	*P*	Unadjusted Odd Ratio (OR) [95%CI]	*P*	Adjusted Odd Ratio (AOR) [95%CI]	*P*
*Community level factors*								
Type of residence								
Urban	1.00				1.00			
Rural	1.56 (1.24,1.96)	<0.001			2.03 (1.74,2.38)	<0.001		
Geopolitical Zones								
North Central	1.00		1.00		1.00		1.00	
North East	2.20 (1.72,2.83)	<0.001	1.92 (1.49,2.46)	<0.001	2.13 (1.74,2.59)	<0.001	1.53 (1.26,1.87)	<0.001
North West	4.62 (3.74,5.71)	<0.001	4.67 (3.72,5.86)	<0.001	3.19 (2.67,3.83)	<0.001	2.74 (2.29,3.27)	<0.001
South East	0.58 (0.42,0.81)	0.001	0.54 (0.39,0.74)	<0.001	0.49 (0.39,0.62)	<0.001	0.49 (0.39,0.62)	<0.001
South West	0.73 (0.54,0.98)	0.039	0.78 (0.58,1.05)	0.098	0.57 (0.46,0.71)	<0.001	0.65 (0.52,0.80)	<0.001
South South	0.94 (0.73,1.22)	0.662	0.98 (0.75,1.29)	0.904	0.74 (0.61,0.90)	0.003	0.98 (0.81,1.19)	0.832
*Socio*-*demographic factors*								
Wealth Index								
Poorest	1.00		1.00		1.00		1.00	
Poor	0.64 (0.52,0.79)	<0.001	0.89 (0.77,1.04)	0.151	0.65 (0.55,0.77)	<0.001	0.87 (0.76,0.98)	0.024
Middle	0.39 (0.31,0.51)	<0.001	0.91 (0.75,1.09)	0.321	0.40 (0.33,0.49)	<0.001	0.77 (0.66,0.89)	<0.001
Rich	0.36 (0.29,0.46)	<0.001	0.72 (0.58,0.88)	0.002	0.29 (0.24,0.35)	<0.001	0.62 (0.52,0.73)	<0.001
Richest	0.22 (0.17,0.29)	<0.001	0.59 (0.47,0.76)	<0.001	0.18 (0.15,0.23)	<0.001	0.45 (0.37,0.55)	<0.001
Maternal working status								
Non-working	1.00				1.00		1.00	
Working (past 12 months)	1.22 (0.88,1.68)	0.232			1.59 (1.29,1.95)	<0.001	1.54 (1.23,1.93)	<0.001
Maternal education								
No education	1.00				1.00			
Primary	0.42 (0.35,0.51)	<0.001			0.46 (0.41,0.53)	<0.001		
Secondary and above	0.31 (0.26,0.37)	<0.001			0.28 (0.24,0.32)	<0.001		
Paternal occupation								
Non agriculture	1.00				1.00			
Agriculture	1.08 (0.75,1.54)	0.695			1.19 (0.89,1.56)	0.226		
Not working	0.76 (0.62,0.94)	0.010			0.72 (0.62,0.84)	<0.001		
Paternal education								
No education	1.00		1.00		1.00			
Primary	0.45 (0.37,0.55)	<0.001	0.87 (0.71,1.06)	0.175	0.48 (0.42,0.55)	<0.001		
Secondary and above	0.32 (0.27,0.38)	<0.001	0.76 (0.64,0.91)	0.003	0.33 (0.29,0.37)	<0.001		
Marital status								
Currently married	1.00				1.00			
Formerly married^+^	0.56 (0.34,0.94)	0.028			0.78 (0.59,1.04)	0.092		
Mother’s literacy								
Can’t read at all	1.00				1.00			
Can read	0.43 (0.36,0.50)	<0.001			0.37 (0.33,0.42)	<0.001		
*Environmental factors*								
Source of drinking water								
Unimproved	1.00				1.00			
Improved	0.82 (0.70,0.96)	0.016			0.74 (0.65,0.85)	<0.001		
*Media factors*								
Reading newspaper								
Yes	1.00				1.00		1.00	
No	1.88 (1.52,2.34)	<0.001			2.53 (2.16,2.97)	<0.001	1.18 (1.02,1.38)	0.043
Listening to radio								
Yes	1.00				1.00			
No	1.56 (1.36,1.78)	<0.001			1.64 (1.47,1.83)	<0.001		
Watching TV								
Yes	1.00				1.00		1.00	
No	2.34 (2.02,2.72)	<0.001			2.60 (2.30,2.94)	<0.001	1.38 (1.23,1.55)	<0.001
*Proximate determinants*								
*Maternal factors*								
Mother’s age								
15–24 years	1.00				1.00			
25–34 years	0.79 (0.67,0.92)	0.003			0.77 (0.69,0.87)	<0.001		
35–49years	1.03 (0.85,1.24)	0.780			0.85 (0.75,0.96)	0.009		
Mother's age at birth								
< 20 years	1.00				1.00			
20–29 years	0.63 (0.51,0.78)	<0.001			0.62 (0.54,0.72)	<0.001		
30–39 years	0.61 (0.49,0.76)	<0.001			0.57 (0.49,0.66)	<0.001		
40 and above	0.86 (0.63,1.17)	0.331			0.69 (0.56,0.86)	0.001		
Mother’s BMI								
< 18.5	1.00				1.00		1.00	
18.5–25	0.86 (0.66,1.13)	0.273			0.73 (0.61,0.88)	0.001	0.99 (0.84,1.17)	0.913
25 and above	0.50 (0.37,0.69)	0.010			0.38 (0.31,0.47)	<0.001	0.79 (0.66,0.95)	0.012
*Delivery factors*								
Type of delivery assistance								
Health professional	1.00				1.00		1.00	
Traditional birth attendant	2.81 (2.32,3.41)	<0.001			2.67 (2.31,3.09)	<0.001	1.19 (1.05,1.36)	0.007
Relatives or other	2.09 (1.71,2.56)	<0.001			2.37 (2.07,2.73)	<0.001	1.11 (0.98,1.24)	0.089
No one	4.18 (3.37,5.18)	<0.001			4.12 (3.49,4.86)	<0.001	1.24 (1.07,1.44)	0.004
Combined Place and mode of delivery								
Home delivery	1.00				1.00			
Health facility with no Caesarean	0.38 (0.32,0.45)	<0.001			0.37 (0.33,0.41)	<0.001		
Health facility with Caesarean	0.31 (0.18,0.54)	<0.001			0.27 (0.19,0.39)	<0.001		
*Pre*/*Post*-*delivery factors*								
Antenatal clinic visits								
None	1.00				1.00			
1–3.	0.68 (0.54,0.85)	0.001			0.65 (0.54,0.78)	<0.001		
4 and above	0.41 (0.34,0.49)	<0.001			0.39 (0.34,0.46)	<0.001		
Timing of postnatal check-up								
No postnatal check-up	1.00				1.00			
0–2 days	0.44 (0.36,0.53)	<0.001			0.47 (0.41,0.54)	<0.001		
Delayed	0.32 (0.24,0.43)	<0.001			0.37 (0.31,0.45)	<0.001		
Currently breastfeeding								
No	1.00				1.00			
Yes	1.02 (0.86,1.21)	0.816			1.04 (0.95,1.15)	0.384		
Duration of breastfeeding								
upto 12 months	1.00		1.00		1.00		1.00	
> 12 months	2.27 (1.98,2.61)	<0.001	1.19 (1.01,1.42)	0.043	2.50 (2.22,2.82)	<0.001	3.28 (2.95,3.65)	<0.001
*Child factors*								
Birth order								
First-born	1.00				1.00			
2nd-4th	1.02 (0.84,1.24)	0.845			1.06 (0.95,1.19)	0.295		
5 or above	1.52 (1.24,1.86)	<0.001			1.43 (1.26,1.62)	<0.001		
Preceding birth interval								
No previous birth	1.00				1.00			
< 24 months	1.14 (0.89,1.47)	0.291			1.44 (1.26,1.65)	<0.001		
> 24 months	1.25 (1.04,1.51)	0.018			1.17 (1.04,1.31)	0.008		
Gender								
Male	1.00		1.00		1.00		1.00	
Female	0.79 (0.69,0.91	0.001	0.69 (0.63,0.77)	<0.001	0.86 (0.81,0.94)	<0.001	0.81 (0.75,0.87)	<0.001
Perceived size of baby								
Small	1.00		1.00		1.00		1.00	
Average	0.65 (0.54,0.71)	<0.001	0.71 (0.61,0.83)	<0.001	0.72 (0.63,0.83)	<0.001	0.91 (0.81,1.02)	0.118
Large	0.42 (0.34,0.51)	<0.001	0.50 (0.43,0.58)	<0.001	0.53 (0.47,0.61)	<0.001	0.69 (0.61,0.78)	<0.001
Child’s age in months	1.08 (1.06,1.09)	<0.001	1.10 (1.09,1.12)	<0.001	1.01 (1.01,1.02)	<0.001		
Child had diarrhoea recently								
No	1.00		1.00		1.00		1.00	
Yes	1.43 (1.19,1.73)	<0.001	1.22 (0.99,1.49)	0.050	1.46 (1.28,1.67)	<0.001	1.31 (1.16,1.49)	<0.001
Child had fever in last 2 weeks								
No	1.00				1.00			
Yes	1.30 (1.09,1.56)	0.004			1.16 (1.03,1.31)	0.015		

#### Children aged 0–59 months

Children of working mothers were more likely to be stunted compared to children of non-working mothers. Mothers with BMI less than 18.5 were more likely to have stunted children than mothers with BMI greater than 25. Children whose mother had no assistance during delivery were more likely to be stunted than children who were delivered with the assistance of a health professional. Children who were breastfeed for more than 12 months were more prone to being stunted than children who were breastfed for less than 12 months. Male children were more inclined to being stunted than females. Children perceived by their mothers to have been small at time of delivery were more likely to be stunted than children perceived to have been large. Children who had a recent episode of diarrhoea were more likely to be stunted than children who had no recent episode of diarrhoea. Also, children who had a bout of fever 2 weeks preceding the survey were more likely to be stunted than children who had no fever. Children from the poorest households were more likely to be stunted than children from the richest households. Children from households that do not read the newspaper were more likely to be stunted when compared with children from households who read the newspaper. Children who reside in the North West geopolitical zone were more prone to stunting than children who reside in other geopolitical zones.

In the final model, newspaper reading was replaced with watching of television and it was observed that the households which did not watch television were more likely to have stunted children then households that engage in watching television.

### Factors associated with severe stunting

#### Children aged 0–23 months

Table 3 shows that mothers with no education were more likely to have severely stunted children than mothers with primary education and secondary education. Also currently married mothers were more likely to have severely stunted children than formerly married mothers. Babies that were perceived to be small at time of birth by their mothers were more likely to be severely stunted than babies perceived to be large. Children who had a recent episode of diarrhoea were more susceptible to severe stunting than children who had no recent episode of diarrhoea. Likewise male children were more prone to having severely stunted growth than their female counterpart. Children who reside in the North West geopolitical zone were more likely to be severely stunted than children who reside in other geopolitical zones. Babies that were breastfed for more than 12 months were more susceptible to severe stunting than children that were breastfed for less than 12 months. Also children from the poorest household were more likely to be severely stunted than children from the richest household.

**Table 3:** Unadjusted and adjusted odds ratios (OR) (95% CI) for severely stunted children aged 0–23 and 0–59 months

Characteristic	Severely Stunted children 0–23 months				Severely Stunted children 0–59 months			
	Unadjusted Odd Ratio (OR) [95%CI]	*P*	Adjusted Odd Ratio (AOR) [95%CI]	*P*	Unadjusted Odd Ratio (OR) [95%CI]	*P*	Adjusted Odd Ratio (AOR) [95%CI]	*P*
*Community level factors*								
Type of residence								
Urban	1.00				1.00			
Rural	1.85 (1.44,2.36)	<0.001			2.29 (1.87,2.81)	<0.001		
Geopolitical Zones								
North Central	1.00		1.00		1.00		1.00	
North East	2.58 (1.79,3.71)	<0.001	2.03 (1.48,2.79)	<0.001	2.21 (1.73,2.80)	<0.001	1.65 (1.31,2.08)	<0.001
North West	5.59 (4.17,7.49)	<0.001	5.65 (4.23,7.54)	<0.001	3.83 (3.09,4.75)	<0.001	3.45 (2.83,4.19)	<0.001
South East	0.50 (0.28,0.89)	0.021	0.41 (0.26,0.66)	<0.001	0.36 (0.25,0.52)	<0.001	0.40 (0.29,0.55)	<0.001
South West	0.53 (0.33,0.87)	0.011	0.75 (0.50,1.13)	0.173	0.59 (0.44,0.79)	<0.001	0.73 (0.56,0.95)	0.022
South South	0.80 (0.55,1.16)	0.476	0.83 (0.57,1.19)	0.310	0.59 (0.46,0.77)	<0.001	0.79 (0.62,1.01)	0.066
*Socio*-*demographic factors*								
Wealth Index								
Poorest	1.00		1.00		1.00		1.00	
Poor	0.71 (0.56,0.89)	0.004	1.01 (0.85,1.19)	0.941	0.69 (0.57,0.82)	<0.001	0.97 (0.85,1.11)	0.659
Middle	0.36 (0.27,0.49)	<0.001	0.89 (0.72,1.12)	0.344	0.40 (0.33,0.50)	<0.001	0.85 (0.72,0.99)	0.049
Rich	0.35 (0.27,0.47)	<0.001	0.74 (0.57,0.96)	0.026	0.26 (0.21,0.32)	<0.001	0.63 (0.52,0.76)	<0.001
Richest	0.19 (0.14,0.28)	<0.001	0.64 (0.47,0.89)	0.007	0.16 (0.12,0.21)	<0.001	0.46 (0.37,0.58)	<0.001
Maternal working status								
Non-working	1.00				1.00		1.00	
Working (past 12 months)	1.24 (0.79,1.92)	0.343			1.57 (1.23,1.99)	<0.001	1.49 (1.17,1.91)	0.002
Maternal education								
No education	1.00		1.00		1.00		1.00	
Primary	0.33 (0.26,0.43)	<0.001	0.87 (0.71,1.05)	0.148	0.39 (0.33,0.46)	<0.001	0.82 (0.69,0.96)	0.014
Secondary and above	0.27 (0.21,0.34)	<0.001	0.74 (0.59,0.94)	0.013	0.23 (0.19,0.28)	<0.001	0.77 (0.64,0.92)	0.004
Paternal occupation								
Non agriculture	1.00				1.00			
Agriculture	1.07 (0.71,1.62)	0.758			1.12 (0.86,1.45)	0.406		
Not working	0.89 (0.71,1.13)	0.357			0.74 (0.62,0.88)	0.001		
Paternal education								
No education	1.00				1.00		1.00	
Primary	0.43 (0.33,0.54)	<0.001			0.46 (0.39,0.55)	<0.001	0.96 (0.82,1.12)	0.57
Secondary and above	0.31 (0.25,0.38)	<0.001			0.30 (0.26,0.36)	<0.001	0.83 (0.71,0.97)	0.018
Marital status								
Currently married	1.00		1.00		1.00		1.00	
Formerly married^+^	0.31 (0.15,0.64)	0.002	0.43 (0.21,0.89)	0.022	0.47 (0.32,0.71)	<0.001	0.63 (0.44,0.90)	0.011
Mother’s literacy								
Can’t read at all	1.00				1.00		1.00	
Can read	0.38 (0.31,0.46)	<0.001			0.31 (0.27,0.37)	<0.001	0.78 (0.69,0.89)	<0.001
*Environmental factor*								
Source of drinking water								
Unimproved	1.00				1.00			
Improved	0.84 (0.69,1.03)	0.087			0.74 (0.63,0.86)	<0.001		
*Media factors*								
Reading newspaper								
Yes	1.00				1.00			
No	1.78 (1.34,2.36)	<0.001			2.68 (2.16,3.32)	<0.001		
Listening to radio								
Yes	1.00				1.00			
No	1.44 (1.22,1.70)	<0.001			1.64 (1.45,1.85)	<0.001		
Watching TV								
Yes	1.00				1.00			
No	2.38 (1.96,2.89)	<0.001			2.99 (2.57,3.48)	<0.001		
*Proximate determinants*								
*Maternal factors*								
Mother’s age								
15–24 years	1.00				1.00			
25–34 years	0.77 (0.64,0.94)	0.008			0.77 (0.68,0.88)	<0.001		
35–49 years	1.03 (0.83,1.28)	0.768			0.83 (0.72,0.97)	0.016		
Mother’s age at birth								
< 20 years	1.00				1.00			
20–29 years	0.61 (0.47,0.79)	<0.001			0.65 (0.55,0.77)	<0.001		
30–39 years	0.59 (0.45,0.78)	<0.001			0.59 (0.50,0.72)	<0.001		
40 and above	0.84 (0.58,1.22)	0.351			0.72 (0.55,0.95)	<0.001		
Mother’s BMI								
< 18.5	1.00				1.00		1.000	
18.5–25	0.83 (0.61,1.13)	0.239			0.71 (0.58,0.87)	0.001	0.93 (0.78,1.12)	0.451
25 and above	0.46 (0.32,0.67)	<0.001			0.36 (0.29,0.46)	<0.001	0.77 (0.63,0.95)	0.016
*Delivery factors*								
Type of delivery assistance								
Health professional	1.00				1.00			
Traditional birth attendant	3.19 (2.50,4.08)	<0.001			3.16 (2.65,3.76)	<0.001		
Relatives or other	2.43 (1.88,3.14)	<0.001			2.69 (2.25,3.20)	<0.001		
No one	4.77 (3.71,6.13)	<0.001			4.76 (3.94,5.75)	<0.001		
Combined Place and mode of delivery								
Home delivery	1.00				1.00			
Health facility—no Caesarean	0.32 (0.26,0.40)	<0.001			0.32 (0.27,0.38)	<0.001		
Health facility with Caesarean	0.30 (0.15,0.61)	0.001			0.20 (0.12,0.33)	<0.001		
*Pre*/*Post*-*delivery factors*								
Antenatal clinic visits								
None	1.00				1.00			
1–3.	0.69 (0.52,0.89)	0.006			0.62 (0.49,0.77)	<0.001		
4 and above	0.39 (0.32,0.49)	<0.001			0.35 (0.30,0.42)	<0.001		
Timing of postnatal check-up								
No postnatal check-up	1.00				1.00			
0–2 days	0.41 (0.32,0.52)	<0.001			0.42 (0.34,0.50)	<0.001		
Delayed	0.25 (0.16,0.39)	<0.001			0.34 (0.25,0.44)	<0.001		
Currently breastfeeding								
No	1.00				1.00			
Yes	0.74 (0.59,0.92)	0.007			0.89 (0.79,0.99)	0.046		
Duration of breastfeeding								
upto 12 months	1.00		1.00		1.00		1.00	
> 12 months	2.13 (1.82,2.49)	<0.001	2.59 (2.19,3.08)	<0.001	2.29 (1.99,2.63)	<0.001	2.83 (2.48,3.22)	<0.001
*Child factors*								
Birth order								
First-born	1.00				1.00			
2nd-4th	0.95 (0.73,1.24)	0.703			1.03 (0.89,1.19)	0.706		
5 or above	1.39 (1.07,1.81)	0.013			1.44 (1.23,1.69)	<0.001		
Preceding birth interval								
No previous birth	1.00				1.00			
< 24 months	1.01 (0.74,1.39)	0.928			1.46 (1.24,1.72)	<0.001		
> 24 months	1.17 (0.91,1.51)	0.221			1.15 (0.99,1.33)	0.064		
Sex of baby								
Male	1.00		1.00		1.00		1.00	
Female	0.65 (0.55,0.77)	<0.001	0.61 (0.54,0.69)	<0.001	0.83 (0.76,0.91)	<0.001	0.77 (0.71,0.85)	<0.001
Perceived size of baby								
Small	1.00		1.00		1.00		1.00	
Average	0.67 (0.53,0.85)	0.001	0.79 (0.67,0.94)	0.009	0.67 (0.57,0.79)	<0.001	0.84 (0.73,0.96)	0.008
Large	0.42 (0.33,0.54)	<0.001	0.58 (0.49,0.69)	<0.001	0.51 (0.44,0.59)	<0.001	0.68 (0.59,0.78)	<0.001
Child’s age in months	1.07 (1.05,1.08)	<0.001	1.11 (1.10,1.13)	<0.001	1.01 (1.00,1.01)	<0.001		
Child had diarrhoea recently								
No	1.00		1.00		1.00		1.00	
Yes	1.48 (1.17,1.87)	0.001	1.31 (1.03,1.67)	0.026	1.64 (1.39,1.92)	<0.001	1.58 (1.38,1.82)	<0.001
Child had fever in last 2 weeks								
No	1.00				1.00			
Yes	1.17 (0.93,1.47)	0.176			1.10 (0.95,1.27)	0.194		

#### Children aged 0–59 months

Working mothers were more likely to have severely stunted children than non-working mothers. Currently married mothers were more likely to have severely stunted children than formerly married mothers. Mothers with a BMI less than 18.5 were more likely to have children with severely stunted growth than mothers whose BMI is greater than 25. Mothers who cannot read were more likely to have severely stunted children than mothers that can read. Also male children were more likely to be severely stunted when compared with their female counterpart. Babies that were breastfed for more than 12 months were more susceptible to severe stunting than children that were breastfed for less than 12 months.

Babies that were perceived to be small at time of birth are more prone to being severely stunted than large babies. Children that had a recent episode of diarrhoea were more likely to have severely stunted growth than children who had not. Children from the poorest households were more likely to be severely stunted than children from richest households. Children who reside in the North West geopolitical zone were more susceptible to severe stunting when compared with those who reside in other geopolitical zones.

In the final model, we replaced wealth index with paternal education, the result showed that paternal education was associated with severe stunting among children aged 0–59 months. Similarly, we also replaced maternal literacy with maternal education and the result showed that maternal education was associated with severe stunting.

## Discussion

The present paper examines factors associated with stunting and severe stunting among children aged 0–59 months in Nigeria. The main factors associated with stunting in the study were: sex of the child, perceived birth size, children who had diarrhoea, duration of breastfeeding, wealth index and geopolitical zone. Factors associated with severe stunting included: sex of the child, perceived birth size, children who had diarrhoea, wealth index, geopolitical zone and maternal BMI.

In this study, we observed that male children had a significantly higher risk of being stunted and severely stunted than their female counterpart. This gender based health inequality may be as a result of community specific cultures in Nigeria which reflect a historical pattern of preferential treatment of females due to the high value placed on women’s agricultural labor [15]. Also male children tend to be more physically active and expend large amounts of energy which should have been channeled into increasing growth. On the other hand, females are culturally expected to be less active and stay at home with their mothers near food preparation. This finding is consistent with results from other cross-sectional studies carried out in Iran [16], Kenya [17], Indonesia [18], Tanzania [19] and Ghana [20].

We also found that the mother’s perception of the baby’s size at time of birth, which serves as a proxy for birth weight, played a crucial role in determining the growth potential of the baby. Babies that were perceived by their mothers to be small or average in size at birth tended to be more predisposed to having stunted and severely stunted growth when compared with larger babies. This reduced birth size could be due to poor maternal nutrition during the pregnancy period. During this period, the child is entirely dependent on the mother for its nutrition via the placenta, thus any nutrition deprivation from the mother will adversely affect the growth and proper development of the child. Also, it is estimated that intrauterine growth restriction due to maternal undernutrition accounts for 20% of the global burden of child stunting [21]. A similar study carried out in Kenya also showed that children who were of average birth size were 1.4 times more likely to be stunted than children who were large in size [17]. Another study conducted in Pakistan on children less than 24 months of age reveals that children with lower birth weight were 3 times more likely to be stunted than children of the same age group with normal or higher birth weight [22]. However, caution should be taken in interpreting the relationship between the mother’s perceived birth size and stunting in our study as the rationale used by the mothers in estimating the size of their babies is unclear.

This study showed that children from poor households are at a greater risk of being stunted and severely stunted than children from richer households. This may be attributed to the fact that with less income to spend on proper nutrition, children from underprivileged households are more prone to growth failure due to insufficient food intake, higher risk of infection as well as lack of access to basic health care services. This finding is supported by a study carried out in Zambia where children from poorer households reported a lower nutritional status than those from richer households [23]. Similar results were obtained from cross-sectional studies conducted in Iran [16] and Nepal [24]. Therefore, to improve child health in poor households, an establishment of properly functioning economic and financial structures which supports children from underprivileged households is needed so as to improve food security and access to basic health care services.

The geopolitical region in which the child resides has a part to play in the likelihood of children to being stunted and severely stunted. It was observed that children that reside in the Northern region of the country exhibit a greater tendency to being stunted when compared with children in the Southern region of the country. This finding may be attributed to cultural beliefs and practices unique to the region which label some nutrient-rich food types as taboo thereby resulting to less consumption of these food types by the growing child, these may have an adverse effect on the nutritional status of the child [25]. Also, people residing in the northern region are mostly farmers; they depend largely on agriculture and livestock products. However, an on-going terrorism insurgency has left the northern farmlands devastated as large areas meant for agriculture are cut-off thus decreasing the region’s agricultural potential and leading to an increase in food insecurity, hence affirming the Nigerian government recent concerns with the level of malnutrition in the Northern region of the country [26]. This is also consistent with a cross sectional survey carried out in Ghana which showed that geographical region was significantly related to stunting and children from the Eastern Region of the country were more likely to be stunted than children from the Western Region which in that study was the reference group [20].

In this study, the duration of breastfeeding was found to be significantly associated with stunting and severe stunting. Children who were breastfed for more than 12 months were more likely to be stunted and severely stunted than those breastfed for less than 12 months. This might be as a result of cultural influences, exclusive breastfeeding status, socioeconomic dynamics, time of initiation of complementary feeding, quality of complementary feed and mother’s educational status [27, 28]. A recent Nepalese study also reported that prolonged breastfeeding (more than 12 months) led to increased risk of stunting and severe stunting among Nepalese children [24].

Children who had a diarrhoeal episode 2 weeks prior to the survey were more prone to being stunted and severely stunted than children who did not have such an episode. This finding may be attributed to the fact that diarrhoea noted 2 weeks prior to the survey may be indicative of a chronic or recurring diarrhoea problem which results from inadequate dietary intake and leads to poor nutritional status through reduced appetite, increased catabolism, impaired intestinal absorption, and direction of essential nutrients away from growth and towards immune response thereby leading to growth failure [29]. A pooled analysis of nine studies with data covering a 20-year period and five countries confirmed the effect of diarrhoea on stunting and supports the hypothesis that the odds of stunting increases multiplicatively with each diarrhoeal episode[8]. Furthermore, a recent study conducted in South Ethiopia reported that the presence of diarrhoea in under-5 year old children 2 weeks prior to the survey was significantly associated with stunting [30].

The type of delivery assistance received was found to be a significant risk factor for stunting. Children who were delivered without any assistance from health professionals were found to be significantly more likely to be stunted compared to those who were delivered with assistance from health professionals. This could be related to the fact that new mothers received valuable nursing information from the health professionals who assisted in the delivery of their baby. Similar findings were reported in recent cross-sectional studies conducted in Nepal [24] and India [31] which recorded a higher rate of stunting and severe stunting among home delivery children compared to health institution delivery children.

Children born to educated fathers were less likely to be stunted when compared with children born to uneducated fathers. Also, children who were born to educated mothers and breastfed were less likely to be severely stunted when compared with children who were born to uneducated mothers and not breastfed. This emphasizes a positive relationship between breastfeeding and parent education in the development of a nutritionally balanced child. This finding is supported by previous cross-sectional studies conducted in Nepal [24], Cambodia [32] and Bangladesh [33].

This study also revealed a predictive impact of maternal BMI on stunting after controlling for a range of child and maternal factors. The mother’s BMI was found to be significantly associated with stunting and severe stunting in under-5 year old children. Mothers with a BMI less than 18.5 kg/m^2^ were significantly more likely to have stunted children than mothers with BMI of 25 kg/m^2^ or above. Past studies show that maternal BMI is an important risk factor associated with poor intrauterine growth and low birthweight; these in turn are known to be determinants of stunting and severe stunting in early childhood [29]. A similar cross-sectional study conducted in Bangladesh reported that mother’s BMI which is an indicator of the mother’s nutritional status was significantly associated with severe as well as moderate stunting [33]. The impact of mother’s nutritional status begins *in utero* and continues for at least the first 6 months of post-natal life when the infant is totally dependent on the mother for all its nutrient supply.

This study is population-based with a large sample size which achieved a 96% and 98% response rate for children and women respectively. It applied appropriate statistical adjustments to data obtained from a nationally representative survey and was able to identify the most vulnerable subpopulation affected by stunting or severe stunting in a large sample. The study used the 2013 NDHS dataset which is the most recent nationally and internationally recognised data available in Nigeria thereby giving relevance to the study. Hence findings from this study can be generalised to the entire Nigerian population.

However, due to the cross-sectional nature of the study design, this paper is limited in its ability to establish a causal relationship between the observed risk factors and stunting/severe stunting. Also, despite the use of a comprehensive set of variables in our analysis, the effect of residual confounding as a result of unmeasured co-variates such as household food security, parent’s height and father’s BMI could not be ruled out.

This study is useful for public health planning and identifying the underlying factors associated with stunting and severe stunting in order to assist in the proper allocation of health resources. It will also assist the Nigerian government in developing and implementing appropriate nutrition programs aimed at improving maternal and child nutrition at both the individual and community levels most especially in Northern Nigeria and in the low socioeconomic strata.

## Conclusion

This study shows that stunting and severe stunting results from a complex interaction of factors. Hence at the individual level, interventions to prevent stunting and severe stunting should focus on improving women’s nutrition to reduce low birth size, improving household hygiene to reduce diarrhoea and on promotion of appropriate complementary food and feeding practices. At the community level, interventions using cash transfer programmes especially among uneducated mothers of low socioeconomic status are needed and such intervention should focus on mothers residing in the Northern geopolitical zones of Nigeria. This intervention strategies will align Nigeria with the WHO global nutrition target of achieving a 40% reduction in the number of stunted children under 5 years by 2025 [5].
